# Society Meetings

**Published:** 1910-09

**Authors:** 


### SOCIETY MEETINGS

The International Congress of School Hygiene, which recently held its triennial session in Paris, will meet in Buffalo in 1913. Dr. Francis E. Fronczak, health commissioner, who represented Buffalo at the Paris Congress only recently closed, succeeded in obtaining a favorable vote to hold the congress here three years hence. This is a notable achievement and reflects credit upon the Commissioner of Health, since invitations had been extended to the congress to meet at Rome, Budapest, Cologne, Prague, Brussels and other cities of Europe.

The American Association of Clinical Research will hold its second annual meeting in Boston, September 28 and 29, 1910. Contributions on researches in medicine and surgery, in prophylactic and anaphylactic medicine, in mental medicine, in radiotherapeutics, in metabolism, and the like, are promised. There will also be a public meeting. The general secretary is Dr. James Krauss, 419 Boylston St., Boston.

